# The quality of care of the dying in hospital—next-of-kin perspectives

**DOI:** 10.1007/s00520-020-05465-2

**Published:** 2020-05-09

**Authors:** Maria Heckel, Annika R. Vogt, Stephanie Stiel, Johannes Radon, Sandra Kurkowski, Swantje Goebel, Christoph Ostgathe, Martin Weber

**Affiliations:** 1 Department of Palliative Medicine, Universitätsklinikum Erlangen, Friedrich-Alexander-Universität Erlangen-Nürnberg (FAU), Krankenhausstraße 12, 91054 Erlangen, Germany; 2 Interdisciplinary Palliative Care Unit, III. Department of Medicine, University Medical Center of the Johannes Gutenberg University of Mainz, Langenbeckstr. 1, 55131 Mainz, Germany; 3 Institute for General Practice, Hannover Medical School, Carl-Neuberg-Straße 1, 30625 Hanover, Germany

**Keywords:** Quality, Death, Palliative care, Caregivers, Outcome assessment (healthcare), Surveys

## Abstract

**Purpose:**

Providing high-quality care for the dying is essential in palliative care. Quality of care can be checked, compared, and improved by assessing responses from bereaved next-of-kin. The objectives of this study are to examine quality of care in the last 2 days of life of hospitalized patients considering specific aspects of their place of care.

**Methods:**

The “Care of the Dying Evaluation” (CODE^™^) questionnaire, validated in German in 2018 (CODE-GER), examines quality of care for the patient and support of next-of-kin, allocating values between 0 (low quality) and 4 (high quality). The total score (0–104) is divided into subscales which indicate support/time given by doctors/nurses, spiritual/emotional support, information/decision-making, environment, information about the dying process, symptoms, and support at the actual time of death/afterwards. Next-of-kin of patients with an expected death in specialized palliative care units and other wards in two university hospitals between April 2016 and March 2017 were included.

**Results:**

Most of the 237 analyzed CODE-GER questionnaires were completed by the patient’s spouse (42.6%) or children (40.5%) and 64.1% were female. Patients stayed in hospital for an average of 13.7 days (3–276; SD 21.1). Half of the patients died in a specialized palliative care unit (50.6%). The CODE-GER total score was 85.7 (SD 14.17; 25–104). Subscales were rated significantly better for palliative care units than for other wards. Unsatisfying outcomes were reported in both groups in the subscales for information/decision-making and information about the dying process.

**Conclusion:**

The overall quality of care for the dying was rated to be good. Improvements of information about the dying process and decision-making are needed.

**Trial registration:**

DRKS00013916

**Electronic supplementary material:**

The online version of this article (10.1007/s00520-020-05465-2) contains supplementary material, which is available to authorized users.

## Introduction

Quality is a central aspect of end-of-life care at home and in an institutional setting; it should therefore be measured to ensure best services and support in the long term. The majority of patients want to spend their last phase of life at home [1–5], but despite this a high proportion of deaths occur in hospitals in most Western European countries [6, 7]—including Germany [8]. As such, death in hospital is a common occurrence and dying patients are treated in almost all hospital settings. The density of specialized palliative care services in hospitals differs among Western European countries [3, 9]. In institutions where such services are available, patients with complex needs receive additional support from specialized hospital support teams or are transferred to a palliative care unit (PCU). In a PCU, death is a frequent and often predictable event [10]. In curative settings, death might be perceived as an unwanted event, which may affect the care of patients and next-of-kin.

Quality of end-of-life care can be measured by assessing responses from informal carers or health professionals [11, 12]. In palliative care, next-of-kin are perceived as a “unit of care” along with the patient [13]. Consequently, next-of-kin may be able to share valuable information about their perception of the quality of care. Several studies on outcomes for the care of the dying have been published, including an assessment of the next-of-kin’s satisfaction with palliative care services [14–16], quality of care [17, 18], patients’ symptom distress [19], and quality estimation of the dying process [12, 20]. A systematic review of psychometric properties of tools measuring quality of death, dying, and care was recently published [21]. Currently, only two instruments for assessing end-of-life care exist in German. The Quality of Dying and Death Questionnaire – German Version (QoDD-D) [11, 12] examines quality of life and considers the circumstances of dying. Care of the Dying Evaluation (CODE^™^) is based on the “Evaluating Care and Health Outcomes – for the Dying” (ECHO-D) [18] and—unlike the QoDD-D—aims at assessing quality of care and support for next-of-kin corresponding to the concept of palliative care in hospitals. It has recently been validated in German (CODE-GER) by the authors’ working group (validation manuscript in review process, reference will be added).

## Research question

The dataset that was originally collected and prepared for the validation study of the CODE-GER was used to perform a secondary analysis on (i) the quality of care for the dying assessed by next-of-kin of patients who died on different wards of two German hospitals and (ii) the differences and similarities in quality of care between patient groups according to place of care.

## Methods

Participating next-of-kin were defined as loved ones, relatives, friends, or other proxies who were in close contact/relationship with the patient and personally visited the patient in hospital in his/her last 2 days of life. This study was registered in the German Clinical Trials Register (DRKS00013916, https://www.drks.de) and is reported according to the STROBE Reporting Guideline [22].

Between April 2016 and March 2017, 1714 deaths occurred on participating wards (internal, neurological, and palliative medicine) in two study centers (Mainz, Erlangen; Germany). Deceased patients were included if they were at least 18 years of age and had stayed in the hospital ward where they died for at least 3 days, death was expected based on the physician’s judgment, and the cause of death was not sudden in nature (validation manuscript in review process, reference will be added). Data necessary to screen for inclusion and exclusion criteria as well as contact addresses of bereaved next-of-kin were extracted through a hospital information system report, from the patient records, and proceeded by the centers research team in accordance with procedures in the participating wards and data protection regulations.

For participation of next-of-kin, an opt-in strategy was used. Following the advice by data protection supervisors, next-of-kin were approached via postcard and in responding actively gave their consent to be contacted for study purposes. The consortium members carefully decided on the time of contact to bereaved next-of-kin, based on experience from previous studies [23]. A data custodian handled the dispatch and return, so that the data from the pseudonymized questionnaires could not be associated with patients by the scientists. No incentives were provided for participation.

After applying the inclusion criteria, the next-of-kin of 914 patients were invited by mail from the center’s researchers 8 to 16 weeks after the patients’ death. A repeat invitation to participate in the study was sent 4 weeks later if a reply had not been received. To assure an inter-rater population during the validation study, additional other next-of-kin (*n* = 223) that had been proposed nominated by participating next-of-kin were contacted. Overall 1137 next-of-kin were contacted, out of which 563 (49.5%) responded to the invitation; *n* = 130 declined participation, and *n* = 433 consented to telephone contact for further information.

During the telephone call, a further 14 next-of-kin were excluded due to insufficient language knowledge (*n* = 1), lack of contact to the patient at hospital in the last 2 days of life (*n* = 3), or self-reported emotional distress (*n* = 10). Another 33 declined due to personal reasons. The remaining consenting next-of-kin (*n* = 386) received the study material by mail. They were contacted by telephone to make sure the study material had been received and to provide support if they had not returned the study material 3 to 4 weeks after initial dispatch. In total, 317 participants (82.1%) returned a completed CODE questionnaire. The final response rate of contacted next-of-kin was 27.9% (317/1137). Six questionnaires were excluded due to missing informed consent statements; a further 26 questionnaires were excluded due to high rates of missing data (> 15%). For the present analysis, a further 48 questionnaires were excluded as they originated from inter-rater participants which was only relevant for the validation study. In total, *n* = 237 questionnaires were included in the present analysis.

### CODE questionnaire

The CODE-GER study examined 7 subscales including 26 items (see Table 2) through principal component analysis. The study demonstrated good psychometric properties: internal consistency, α = 0.86; inter-rater reliability, ICC (1,1) = 0.79; and retest reliability, ICC (2,1) = 0.85. The rating scales differed between questions from binary scales up to 5-point Likert scales (see Table 2 for details), with possible values between 0 and 4.

For each subscale, scores of the included items were added to a subscale score, which in turn were summed up to the total score (0 to 104); higher values indicate a higher quality of care.

Two items from the original CODE questionnaire (addressing “cleanliness of the ward area” and “right place for the patient to die”) were not included and are maintained as optional items in CODE-GER. The “right place for the patient to die” item is reported here as an overall item.

Two further questions assess overall impressions of end-of-life care: an estimation of the amount of time that the patient was treated with respect and dignity, and to what extent the next-of-kin felt adequately supported during the last 2 days of the patient’s life. One overall question examines the respondents’ willingness to recommend the ward to friends or family and was reintroduced from an older version of CODE^™^.

Socio-demographic data on the participant and the patient, and further health/disease-related information on the patient were provided by next-of-kin in the questionnaire.

Space for free text allowed the respondents to give feedback on particular items and the entire questionnaire. If participants provided reasons within the free texts for ticking “do not know” or leaving out the answer to a question, these explanations were included in the “Results” section. The same free texts were included in the “Results” section to aid the interpretation of perceived low quality of care aspects and analyze the potential for improvement.

### Data preparation and analysis

Missing values were imputed via expectation–maximization for interval variables and the modus score of corresponding items for categorical variables; a proportion of up to 15% missing items was tolerated. The authors decided on the 15% threshold according to the missing patterns of the dataset and to similar questionnaires such as the QoDD-D [11, 12], considering the risk of bias due to imputation and the number of questionnaires excluded. Questionnaires with higher missing rates were excluded from analysis. Missing rates are presented for transparency reasons (see Table 2). Missing data of overall items were not imputed. Descriptive statistics were calculated, including mean values and standard deviation (SD) within subscales, frequencies of single items, and missing values.

Group comparisons for quality of care are presented for patients who died at a PCU and patients who died at other wards. *T* tests were used to compare the mean subscale scores and sum score of subscales (total score) for the groups.

Results were considered significant if *p* < 0.05.

Multiple linear regressions were used to find out if group differences were predicted by other variables than place of death.

The analysis was performed using the statistics software SPSS 21 (SPSS Inc., Chicago, IL, USA) developed by IBM [24].

Free texts were analyzed based on the qualitative content analysis approach by Mayring [25] and using the MAXQDA software for qualitative analysis [26].

## Results

### Study population

#### Patients

Half of the deceased patients were between 60 and 79 years old (51.5%) and 50.6% were male. 2.5% of patients were not of German nationality (nationality not stated by 3% of participants). The average length of stay was 13.7 days (range 3–276, SD 21.1). Cancer diagnosis was stated for 56.5% of patients. Half of the patients died in a specialized PCU (50.6%). Those who died at another ward (49.4%) were hospitalized on a normal ward (*n* = 60) or an intensive care unit (*n* = 57).

#### Next-of-kin

Most participants were between 50 and 69 years old (52.7%). 64.6% of respondents were female. Few respondents (2.0%) stated other nationalities than German (nationality not stated by 8.1% of participants). Most of the participants were patients’ spouses (42.6%) or children (40.5%). Others (16.9%) were parents, siblings, grandchildren, friends, or neighbors.

Respondents returned the CODE questionnaire after an average of 124 days (68–354 days, median 114 days) after the patient’s death.

### Quality of care—total score and comparing subscales

The mean CODE-GER total score was 85.7 (range 25–104, SD 14.17). Only 9 questionnaires (3.8%) scored less than 50% of the total score. The mean scores of the subscales and the percentage of their maximum values are presented to enable comparisons between subscales (Table 1). The subscales “information about dying process,” “symptom presence,” and “information and decision-making” showed the lowest mean values (70.5, 70.8, 71.9); the subscales “support and time doctors and nurses” and “support at actual time of death and afterwards” (91.1, 91.8) were rated highest. All subscales have negative skewness values, showing that the distribution is left-skewed and not normally distributed. Participants tend to estimate the quality better than a normal distribution would suggest.

**Table 1:** Subscales comparison using percentage of maximum, mean, SD, percentiles, skewness

Subscale	Values according to scale range			Percentage of maximum values			
	Mean score (scale range)	SD	95% CI	Mean score	SD	95% CI	skewness
(1) Support and time of doctors and nurses	32.8 (0–36)	4.7	32.2–33.4	91.1	13.1	89.3–92.8	− 2.592
(2) Spiritual and emotional support	10.0 (0–12)	2.6	9. 7–10.3	83.4	22.0	80.7–86.2	− 1.531
(3) Information and decision-making	11.5 (0–16)	4.2	11.0–12.1	71.9	26.3	68.6–75.3	− 0.904
(4) Environment	7.1 (0–8)	1.6	6.9–7.3	88.3	19.5	85.8–90.8	− 2.058
(5) Information about dying process	8.4 (0–12)	4.1	7.9–9.0	70.5	34.3	66.0–74.7	− 0.819
(6) Symptom presence	8.5 (0–12)	3.0	8.1–8.9	70.8	24.5	67.6–73.7	− 0.575
(7) Support at actual time of death and afterwards	7.3 (0–8)	1.5	7.2–7.5	91.8	18.7	89.3–94.0	− 3.111

**Fig. 1:**
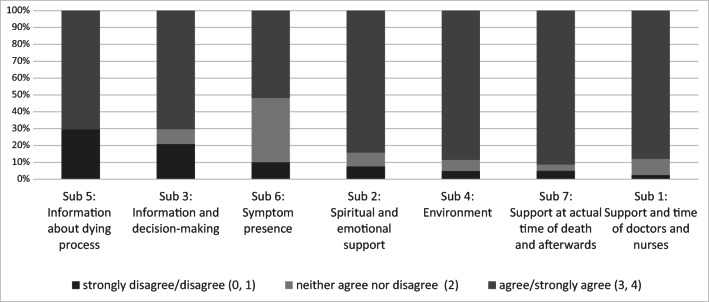
Frequencies of answers in percent per subscale; legend: subscales sorted according to the amount of values 0 and 1

Frequencies of answers give a more detailed insight into the measured quality of care subscales (see Fig. 1). The highest values (3–4) were most often reached in subscale 7 “support at actual time of death and afterwards.”

For the subscales “information about dying process,” “information and decision-making,” and “symptom presence,” up to 29.5% of respondents rated lower values (0–1), thus indicating aspects of care in need of improvement (see Fig. 1). The frequencies of answers to the single items are shown in Table 2.

**Table 2:** Items per subscales, scales, and subscale scores

Subscale (min.–max. subscale scores)	Item^a^	Possible answer options	Frequencies of answers (imputed dataset) (%)	Percentage of missing before imputation
(1) Support and time of doctors and nurses (0–36)	There was enough help available to meet his/her personal care needs, such as washing, personal hygiene, and toileting needs. (1)	Strongly agree (4)	81.0	2.5
		Agree (3)	12.7	
		Neither agree nor disagree (2)	3.8	
		Disagree (1)	2.1	
		Strongly disagree (0)	0.4	
	There was enough help with nursing care, such as giving medicines and helping him/her find a comfortable position in bed. (2)	Strongly agree (4)	83.1	0.0
		Agree (3)	11.4	
		Neither agree nor disagree (2)	3.0	
		Disagree (1)	2.5	
		Strongly disagree (0)	0.0	
	The nurses had time to listen and discuss his/her condition with me. (7)	Strongly agree (4)	67.1	0.4
		Agree (3)	22.8	
		Neither agree nor disagree (2)	5.5	
		Disagree (1)	4.2	
		Strongly disagree (0)	0.4	
	The doctors had time to listen and discuss his/her condition with me. (8)	Strongly agree (4)	68.4	2.5
		Agree (3)	22.4	
		Neither agree nor disagree (2)	3.0	
		Disagree (1)	5.1	
		Strongly disagree (0)	1.3	
	Did you have confidence and trust in the nurses who were caring for him/her? (5)	Yes, in all of them (4)	82.7	0.8
		Yes, in some of them (2)	17.3	
		No, not in any of the nurses (0)	0.0	
	Did you have confidence and trust in the doctors who were caring for him/her? (6)	Yes, in all of them (4)	83.1	2.1
		Yes, in some of them (2)	15.2	
		No, not in any of the doctors (0)	1.7	
	In your view, did the doctors and nurses do enough to help relieve the pain? (10)	Yes, all of the time (4) Not applicable, s/he was not in pain (4)	93.7	1.3
		Yes, some of the time (2)	5.9	
		No, not at all (0)	0.4	
	In your view, did the doctors and nurses do enough to help relieve the restlessness? (12)	Yes, all of the time (4) Not applicable, s/he was not restlessness (4)	82.3	2.5
		Yes, some of the time (2)	16.0	
		No, not at all (0)	1.7	
	In your view, did the doctors and nurses do enough to help relieve the “noisy rattle” to his/her breathing? (14)	Yes, all of the time (4) Not applicable, s/he had no noise rattle (4)	80.6	4.6
		Yes, some of the time (2)	16.9	
		No, not at all (0)	2.5	
(2) Spiritual and emotional support (0–12)	How would you assess the overall level of emotional support given to you by the healthcare team? (19)	Excellent (4)	59.9	0.8
		Good (3)	31.6	
		Fair (1)	6.3	
		Poor (0)	2.1	
	Overall, his/her religious or spiritual needs were met by the healthcare team. (20)	Strongly agree (4)	57.8	10.1
		Agree (3)	23.6	
		Neither agree nor disagree (2)	12.7	
		Disagree (1)	3.8	
		Strongly disagree (0)	2.1	
	Overall, my religious or spiritual needs were met by the healthcare team. (21)	Strongly agree (4)	61.2	10.5
		Agree (3)	18.1	
		Neither agree nor disagree (2)	12.2	
		Disagree (1)	5.9	
		Strongly disagree (0)	2.5	
(3) Information and decision-making (0–16)	During the last 2 days, how involved were you with the decisions about his/her care and treatment? (15)	Very involved (4)	60.8	0.4
		Fairly involved (2)	26.2	
		Not involved (0)	13.1	
	Did any of the healthcare team discuss with you whether giving fluids through a “drip” would be appropriate in the last 2 days of life? (16)	Yes (4)	56.5	1.3
		No (0)	43.5	9.7 (do not know)
	Would a discussion about the appropriateness of giving fluids through a “drip” in the last 2 days of life have been helpful? (17)	No (4) Not applicable, we had these types of discussions (4)	79.3	7.6
		Yes (0)	20.7	
	Did the healthcare team explain his/her condition and/or treatment in a way you found easy or difficult to understand? (18)	Very easy (4)	39.2	0.8
		Fairly easy (3)	45.1	
		Fairly difficult (2)	9.3	
		Very difficult (1)	1.3	
		They did not explain his/her condition or treatment to me (0)	5.1	
(4) Environment (0–8)	The bed area and surrounding environment was comfortable for him/her. (3)	Strongly agree (4)	69.2	0.8
		Agree (3)	17.7	
		Neither agree nor disagree (2)	8.0	
		Disagree (1)	4.2	
		Strongly disagree (0)	0.8	
	The bed area and surrounding environment had adequate privacy for him/her. (4)	Strongly agree (4)	72.2	0.8
		Agree (3)	17.7	
		Neither agree nor disagree (2)	5.5	
		Disagree (1)	3.8	
		Strongly disagree (0)	0.8	
(5) Information about dying process (0–12)	Before s/he died, were you told s/he was likely to die soon? (22)	Yes (4)	85.2	0.4
		No (0)	14.8	
	Did a member of the healthcare team talk to you about what to expect when s/he was dying (e.g., symptoms that may arise)? (23)	Yes (4)	54.4	2.5
		No (0)	45.6	
	Would a discussion about what to expect when s/he was dying have been helpful? (24)	No (4) Not applicable, we had these types of discussions (4)	71.7	6.8
		Yes (0)	28.3	
(6) Symptom presence (0–12)	In your opinion, during the last 2 days, did s/he appear to have a “noisy rattle” to his/her breathing? (13)	Yes, all of the time (0)	13.1	1.7
		Yes, some of the time (2)	39.7	
		No (4)	47.3	
	In your opinion, during the last 2 days, did s/he appear to be in pain? (9)	Yes, all of the time (0)	8.9	0.8
		Yes, some of the time (2)	33.3	
		No (4)	57.8	
	In your opinion, during the last 2 days, did s/he appear to be restless? (11)	Yes, all of the time (0)	8.4	0.4
		Yes, some of the time (2)	41.4	
		No (4)	50.2	
(7) Support at actual time of death and afterwards (0–8)	I was given enough help and support by the healthcare team at the actual time of his/her death. (25)	Strongly agree (4)	73.0	2.1
		Agree (3)	13.1	
		Neither agree nor disagree (2)	7.6	
		Disagree (1)	3.0	
		Strongly disagree (0)	3.4	
	After s/he had died, did individuals from the healthcare team deal with you in a sensitive manner? (26)	Yes (4)	96.2	2.5
		No (0)	3.8	5.1 (not applicable)
Optional items^b^	In your opinion, how clean was the ward area that s/he was in? (O 1)	Very clean (4)	86.5	0.0
		Fairly clean (2)	12.7	
		Not at all clean (0)	0.8	
	In your opinion did s/he die in the right place? (O 2)	Yes, it was the right place (4)	83.5	2.6 (do not know)
		Not sure (2)	6.8	
		No, it was not the right place (0)	7.2	

^a^Original English wording of items; numbered according to CODE-GER

^b^Items were deleted after psychometric analyses for the final version of CODE-GER

Almost half of the participants (45.6%) stated that they had not been told what to expect when the patient was dying, whereas 52.9% of those participants thought information would have been helpful (29.0% out of 237). Free text answers reflected this sentiment in more detail: Next-of-kin reported how and when the information was given, if it was useful, and the implications of lacking information on their grief: “Up to this day, I still can’t cope with the last breaths. Didn’t know it was so extreme, even though my father wasn’t conscious“. Contrarily, it was also stated that more information about what to expect would have possibly worsened the situation: “I think that would have made it even harder for me.” Others reported whether they had been informed in time about the pending death of the patient and if they were able to be with the patient in his or her last hours of life. Free text suggests the beneficial nature of providing leaflets on the dying process, the wish to be given information about the dying process in absence of the patient and the request for guidance on impending death. “It would have been helpful for me [...] to remind me in this exceptional situation to bring his favourite music, lyrics or prayers [...].”

Furthermore, next-of-kin reported that they were surprised by the “suddenness of the dying process,” although more than 80% of participants answered that they were told that the patient would die soon.

The information was most frequently given by doctors (81.1%) or nurses (27.2%) and in few cases (*n* = 4) by others.

43.6% of participants reported that the team did not discuss with them whether hydration would be appropriate in the last 2 days of life, but 26.2% of them would have found such a conversation helpful.

#### Increased rates of missing values

The patient’s and the next-of-kin’s fulfillment of religious or spiritual needs (10.1% and 10.5% missing) are particularly noteworthy due to increased numbers of missing values (see Table 2).

Reasons for skipping the answer were provided in the free text by statements such as “no religious and spiritual needs of patient or next-of-kin” or “religious and spiritual support of no importance for patient or next-of-kin” or “not belonging to any denomination.”

#### Overall assessment on quality of care of the dying in hospitals

Participants mainly considered that the hospital is the right place for the patient’s end of life (83.5%). 7.2% thought that it was not the right place and 6.8%/2.5% were unsure/did not know. Most participants (87.8%) would (likely or rather likely) recommend the ward and ten participants stated that they did not know or skipped the answer.

Overall, 87.8% of participants felt adequately supported during the patient’s last 2 days of life. Most participants stated that the patient was treated with respect and dignity by the doctors always/most of the time (86.5%). Nurses were perceived by 92.9% of respondents as treating the patient with dignity.

### Comparing quality of care according to place of death

For most subscales and the total score, the quality of care is perceived significantly better by next-of-kin at a PCU than on other wards (see Table 3). Symptom presence was higher for patients at a PCU. The information subscales (3, 5) showed similar values. Next-of-kin of patients on other wards stated that they had not been involved in decisions about treatment and care more often than those from a PCU (14.5%/11.7%). The appropriateness of giving fluids was discussed less frequently on a PCU (42.5%) than on other wards (55.6%); every fifth next-of-kin indicated that a discussion on this issue would have been helpful (PCU 22.5%; other wards: 18.8%). Information that the patient was likely to die soon was missing more frequently on a PCU (17.5%) than on other wards (12%). Almost half of the next-of-kin in both settings stated that the healthcare team did not talk to them about what to expect when the patient was dying (PCU 45.8%; other wards 45.3%). A substantial number of next-of-kin indicated that a discussion about this issue would have been helpful (PCU 33.3%; other wards 23.1%).

**Table 3:** Group comparisons of subscales according to “place of care” (patients who died at a specialized PCU and patients who died at other wards)

Groups		Subscales (mean, SD)							Total
		1 0–36	2 0–12	3 0–16	4 0–8	5 0–12	6 0–12	7 0–8	Sum score 0–104
Place of Death	PCU (*n* = 120)	33.89 (2.92)	10.74 (1.83)	11.49 (4.15)	7.59 (0.76)	8.13 (4.34)	7.93 (2.98)	7.74 (0.69)	87.51 (10.32)
	Other wards (n-117)	31.70 (5.88)	9.26 (3.10)	11.53 (4.30)	6.53 (1.95)	8.78 (3.87)	9.08 (2.79)	6.93 (1.93)	83.83 (17.10)
	*T* test (*p*)	*0.000*	*0.000*	0.944	*0.000*	0.223	*0.002*	*0.000*	*0.047*

Statistical significance values (*p* < 0.05) in italic; subscales: (1) support and time of doctors and nurses, (2) spiritual and emotional support, (3) information and decision-making, (4) environment, (5) information about dying process, (6) symptom presence, (7) support at actual time of death and afterwards

Hierarchical multiple regression analysis was conducted for the variables patient’s age (> 60 years vs. < 60 years) and gender, cancer diagnosis, next-of-kin’s age (> 60 years vs. < 60 years) and gender, and relationship to patient (partner vs. others; child vs. others) to identify the best fitting model. The statistically significant model (*F*(6,230) = 2.180, *p* = 0.046) included the variables place of death, cancer diagnosis, relationship to patient (partner vs. others; child vs. others), next-of-kin’s gender, and patient’s age (> 60 years vs. < 60 years). The adjusted *R*^2^ indicated that 2.9% of the variance in the total sum score can be explained by variances in the predictor variables. The analysis suggested that place of death (*ß* = 0.167) was the most influential predictor of the total sum score and next-of-kin’s gender (*ß* = 0.065) was the least influential predictor of the total sum score in the model.

Place of death was shown to be a statistically relevant predictor of the total sum score (*t* = 2.334, *p* = 0.020). Being the patient’s child (*t* = 1.282, *p* = 0.201), patient’s age (*t* = 1.392, *p* = 0.165), being the patient’s partner (*t* = 0.828, *p* = 0.408), cancer diagnosis (*t* = − 0.980, *p* = 0.328), and next-of-kin’s gender (*t* = 1.011, *p* = 0.313) were not statistically relevant predictors of the total sum score (see supplementary Table 1).

Models using the subscale sum scores as criterion variable showed the place of death to be a statistically significant predictor of the scores of subscales 1, 2, 4, and 7 considering other variables. The symptom presence subscale model 6 (see supplementary Table 1) indicated cancer diagnosis to be a statistically significant predictor of the subscale sum score. Place of death was not found to be a statistically significant predictor (see supplementary Table 1). The PCUs in this study had a much higher proportion of patients with cancer diagnosis (76%) than other wards (37%). The findings indicate an effect of cancer diagnosis on symptom presence rather than the place of death.

The majority of next-of-kin reported good general quality of care; however, differences between PCUs and other wards were found (see Table 4).

**Table 4:** Group comparisons of subscales according to “place of care” (patients who died at a specialized PCU and patients who died at other wards)

Groups		Overall items						
		Mean (SD), *n*, missing				Frequency (%)		
		Dying in right place^1^ (range 0–4)	Treated with dignity and respect by doctors^2^ (range 0–4)	Treated with dignity and respect by nurses^2^ (range 1–4)	Recommend ward^3^ (range 0–4)	Support for next-of-kin^4^		
						Yes	No	No answer
Place of death	PCU	3.76 (0.84), *n* = 117, 3 missing	3.95 (0.32), *n* = 108, 12 missing	3.91 (0.36), *n* = 116, 4 missing	3.89 (0.47), *n* = 118, 2 missing	92.5	5.0	2.5
	Other wards	3.37 (1.34), *n* = 114, 3 missing	3.75 (0.77), *n* = 108, 9 missing	3.81 (0.52), *n* = 107, 10 missing	3.31 (1.24), *n* = 109, 8 missing	82.9	13.7	3.4
	*T* test (*p*)	*0.008*	*0.012*	0.091	*0.000*			

Statistical significances (*p* < 0.05) in italic

^1^In your opinion, did s/he die in the right place?

^2^How much of the time was s/he treated with respect and dignity in the last 2 days of life?

^3^How likely are you to recommend our organization to friends and family?

^4^Overall, in your opinion, were you adequately supported during his/her last 2 days of life?

## Discussion

Quality is estimated to be high by the vast majority0 of next-of-kin with few negative exceptions comparable with the results of the original CODE validation study in the UK [27] and others [28]. Subsample comparisons showed higher total scores for PCU patients, even considering other variables. This pattern was the same for the overall impressions. The sensibility for end-of-life care aspects might be higher at PCUs with a holistic quality-of-life approach [29] and training. Environmental and staff-related factors, therapy goals, and expectations of the next-of-kin might differ according to the ward the patient was admitted to, and these factors may change during disease progression. Also, scarce personnel and time resources of other wards could have an impact. Differences in symptom presence showed to be influenced by diagnosis rather than place of death. Next-of-kin of PCU patients rated quality of care significantly higher, except for the subscales relating to information. Notably, improvements are necessary in information and decision-making irrespective of the type of ward. Surprisingly enough, the answers by a substantial part of next-of-kin of patients who died at a PCU indicate information deficits in important aspects, specifically the appropriateness of administering intravenous fluids and the circumstances of the dying process. Despite the inclusion criteria of expected death and 80% of participants who confirmed that they were told that the patient would die soon, free text answers showed that some next-of-kin were surprised by the patient’s death and felt unprepared. Findings from international e.g. qualitative studies that aimed to investigate the family members’ perspectives on dying in hospitals [30, 31] showed similar results. International studies using outcome instruments to measure the quality of care for the dying explicitly in hospital settings in other countries [1, 27, 32] also identified next-of-kin’s perception of deficits in providing information by health professionals and the inclusion in decision-making as well as difficult symptom control. Issues concerning information provision and decision-making are of paramount importance in order to meet patient’s and next-of-kin needs in an appropriate manner. Whereas our study reveals in this regard during the last 2 days of end-of-life care, it is not intended to question staff members’ communication abilities in general. Next-of-kin are in an exceptional situation with a beloved one dying and the transfer of information in these situations might be hampered [33–35]. Previous research on information provision by Verkissen et al. (2019) found a lower percentage (10.7–13.2%) of relatives reporting suboptimal information provision in palliative care settings in Belgium [36] but referred to experiences to the whole length of palliative care guidance. A literature review by Belanger (2011) showed challenges to shared decision-making in palliative care due to delayed decisions and seldom discussion of other treatment options [37]. However, the decision-making experiences refer to the full length of the care trajectory and not the last 2 days as in this study. One major implication for clinical care should therefore be raising sensitivity of PCU staff to the importance of repeated communication on dying who might have become accustomed to the circumstances of the dying process. The respondents’ free text answers in the current study provided additional valuable insights into beneficial interventions such as offering leaflets on the dying process, providing information about the dying process to next-of-kin in absence of the patient, and providing information on arranging the farewell. Information leaflets [38] and conversations should be tailored to next-of-kin needs, comprehension level, and emotional state;, should contain non-medical information as well; and should be offered repeatedly. Better comprehensibility of leaflets could be achieved by patient and public involvement in the design of information material. An important measure—as carried out at the two study site hospitals after receiving the results—should be to involve the hospital quality management and to initiate a process of implementing specific guidelines for care of the dying.

Future research should address communication models and instruments that help to overcome difficulties in information transfer and focus on the content and form of information provided to next-of-kin facing their loved ones dying.

### Strengths and limitations of the study

The major strength of this analysis are the broad insights gained into the under-researched aspect of the quality of care of dying patients in hospitals examining the questionnaires’ subscales, items, and free text answers. Group comparisons have demonstrated the differences and similarities between specialized PCUs and other wards and have helped to identify gaps in the care of the dying in hospital and the next-of-kins’ support. The multiple regression analysis ensures that the differences are not linked to other factors such as patients’ or respondents’ characteristics.

A methodological limitation may be inherent to the opt-in procedure, as primarily next-of-kin that were exceptionally pleased with quality of care or rather dissatisfied may have been more motivated to participate implying a selection bias.

Both study centers are university hospitals and the results are not directly transferable to all hospitals.

The group comparisons did not differentiate between patients on other wards with and without hospital palliative care support teams or between intensive care and acute care.

Although next-of-kin are the preferred sources of patient-centered information as the patient cannot be asked anymore, estimations may be biased by their emotions; thus, selective perception can affect the memorization and the later recall of memory.

Higher missing rates for the emotional and spiritual support items compared with other subscales, for both patients and next-of-kin and irrespective of the type of ward could be inherent in the pluralism of the definition [39] of spirituality.

Findings on quality of care for the dying using the CODE-GER can be utilized to identify gaps in the quality of care, design strategies for improvement, and allow benchmarking between different institutions and settings. Nevertheless, improvement strategies should consider the different therapy aims, specializations, personnel, and financial resources of the settings. The differentiation between quality of care and support for next-of-kin according to the palliative care approach allows measuring outcome and improving both aspects, if needed.

## Conclusions

This study has demonstrated that analyzing CODE-GER contents gives valuable insights into the quality of care during the dying phase in hospitalized patients and identified specific aspects in need of improvement. This can facilitate further quality management and benchmarking initiatives within the hospital and also between institutions.

## Electronic supplementary material

ESM 1 (DOCX 16.2 kb)

## Data Availability

The datasets analyzed during this study are available from the corresponding author on reasonable request.
