# Supplementary material for: The quality of care of the dying in hospital—next-of-kin perspectives
Source: Support Care Cancer. 2020 May 9;28(9):4527–37. doi: 10.1007/s00520-020-05465-2 (PMC7378108; doi:10.1007/s00520-020-05465-2)
Supplement: Supplementary file 1 — (DOCX 16.2 kb) [file 520_2020_5465_MOESM1_ESM.docx]

Supplementary Table 1: Regression models with total sum score as criterion variable and subscales scores as criterion variable

|  | Sum Score Model | Subscale 1 Model | Subscale 2 Model | Subscale 4 Model | Subscale 6 Model | Subscale 7 Model |
| --- | --- | --- | --- | --- | --- | --- |
| Place of death palliative care unit | 4.711* (2.018) | 2.535***(0.672) | 1.549*** (0.365) | 1.033***(0.212) | -0.604 (0.409) | 0.837*** (0.209) |
| Patient’s age >60 years | 3.620 (2.601) | 0.542 (0.908) | 0.499 (0.493) | (0.504) (0.287) | 0.635 (0.552) | -0.078 (0.282) |
| Patient’s gender female | - | -0.235 (0.696) | 0.509 (0.378) | -0.026 (0.220) | -0.250 (0.423) | 0.229 (0.216) |
| Cancer diagnosis | -2.054 (2.096) | -0.494 (0.705) | -0.288 (0.383) | 0.335 (0.223) | -0.960*(0.429) | -0.165 (0.219) |
| Next-of-kin’s age >60 years | 3.620 (2.601) | 0.566 (0.777) | 0.197 (0.422) | 0.203 (0.245) | -0.245 (0.472) | 0.183 (0.241) |
| Next-of-kin female | 1.924 (1.904) | 0.714 (0.713) | 0.061 (0.387) | 0.082 (0.225) | 0.803 (0.434) | 0.391 (0.221) |
| Relationship child | 3.604 (2.812) | -0.005 (0.973) | 0.667 (0.528) | -0.201 (0.307) | -0.053 (0.592) | 0.260 (0.302) |
| Relationship partner | 2.209 (2.668) | 0.063 (0.932) | -0.129 (0.506) | 0.028 (0.295) | 0.904 (0.567) | -0.202 (0.290) |
| Constant | 78.425*** (3.266) | 30.945*** (1.181) | 8.399*** (0.641) | 5.904*** (0.373) | 8.416*** (0.718) | 6.718*** (0.367) |
| Observations | 237 | 237 | 237 | 237 | 237 | 237 |
| Adjusted R² | 0.029 | 0.039 | 0.085 | 0.118 | 0.075 | 0.067 |
| F | (6,230) 2.180 p=0.046 | (8,228) 2.184 p=0.030 | (8,228) 3.727 p<0.001 | (8,228) 4.946 p<0.001 | (8,228) 3.395 p=0.001 | (8,228) 3.122 p=0.002 |

Sign: * <0.05 ** <0.01 *** <0.001

The quality of care of the dying in hospital – Next-of-kin perspectives
Secondary analysis of the CODE-GER validation data set
